# RETRACTION: Effect of Minimally Invasive Versus Conventional Aortic Root Replacement on Transfusion and Postoperative Wound Complications in Patients: A Meta‐Analysis

**DOI:** 10.1111/iwj.70389

**Published:** 2025-03-21

## Abstract

**RETRACTION:** ChenY. , YuW. , JiangY. , GaoJ. , XieD. , YuJ. , LiW. , LiuZ. , and XiongJ. , “Effect of Minimally Invasive Versus Conventional Aortic Root Replacement on Transfusion and Postoperative Wound Complications in Patients: A Meta‐Analysis,” International Wound Journal 21, no. 2 (2024): e14396, 10.1111/iwj.14396.PMC1082460037740672

The above article, published online on 23 September 2023, in Wiley Online Library (http://onlinelibrary.wiley.com/), has been retracted by agreement between the journal Editor in Chief, Professor Keith Harding; and John Wiley & Sons Ltd. Following an investigation by the publisher, all parties have concluded that this article was accepted solely on the basis of a compromised peer review process. The editors have therefore decided to retract the article. The authors did not indicate their agreement with the retraction.

**RETRACTION:** Y. Chen , W. Yu , Y. Jiang , J. Gao , D. Xie , J. Yu , W. Li , Z. Liu , and J. Xiong , “Effect of Minimally Invasive Versus Conventional Aortic Root Replacement on Transfusion and Postoperative Wound Complications in Patients: A Meta‐Analysis,” International Wound Journal 21, no. 2 (2024): e14396, 10.1111/iwj.14396.PMC1082460037740672

The above article, published online on 23 September 2023, in Wiley Online Library (http://onlinelibrary.wiley.com/), has been retracted by agreement between the journal Editor in Chief, Professor Keith Harding; and John Wiley & Sons Ltd. Following an investigation by the publisher, all parties have concluded that this article was accepted solely on the basis of a compromised peer review process. The editors have therefore decided to retract the article. The authors did not indicate their agreement with the retraction.

